# Fournier’s Gangrene: A Coexistence or Consanguinity of SGLT-2 Inhibitor Therapy

**DOI:** 10.7759/cureus.27773

**Published:** 2022-08-08

**Authors:** Tutul Chowdhury, Nicole Gousy, Amulya Bellamkonda, Jui Dutta, Chowdhury F Zaman, Ummul B Zakia, Tasniem Tasha, Priyata Dutta, Padmaja Deb Roy, Adriana M Gomez, Arjun Mainali

**Affiliations:** 1 Internal Medicine, One Brooklyn Health System, Brooklyn, USA; 2 Medicine, American University of Antigua, New York, USA; 3 Medicine, Comilla Medical College, Cumilla, BGD; 4 Internal Medicine, Jahurul Islam Medical College and Hospital, Cedar Lake, USA; 5 Internal Medicine, Sir Salimullah Medical College, Dhaka, BGD; 6 Internal Medicine, Rajshahi Medical College, Rajshahi, BGD; 7 Internal Medicine, Trinity Health, St Joseph Mercy Ann Arbor, Ann Arbor, USA; 8 Medicine, Comilla Medical College, New york, USA; 9 Internal Medicine/Endocrinology, One Brooklyn Health System, Brooklyn, USA

**Keywords:** medication side effects, genitourinary infections, diabetes mellitus, sodium–glucose cotransporter-2 inhibitors, fournier’s gangrene

## Abstract

Background: Sodium-glucose cotransporter-2 inhibitors (SGLT2 inhibitors) are a relatively new class of medications used for the management of type II diabetes mellitus targeting the kidneys. Within the last decade, several warnings have been issued regarding the development of severe genitourinary infections, including necrotizing fasciitis, or Fournier’s gangrene, in those with pre-existing type II diabetes and concomitant use of this drug class.

Objective: The purpose of this review is to highlight and discuss the factors contributing to the development of Fournier’s gangrene, its pathogenesis, and a review of existing literature describing patient outcomes, treatment, and future directions regarding early detection of this complication.

Methods: Articles and studies addressing effective treatment adherence and key factors contributing to Fournier’s gangrene with SGLT2 inhibitors were identified by effective keyword searches in PubMed Central, Google Scholar, and Cochrane, as well as the references found within these articles.

Results: Using the keywords provided, 55 case reports, review articles, and meta-analysis reports written within the last 20 years were utilized as the source of the data presented in this systematic review article.

## Introduction and background

**Figure 1:**
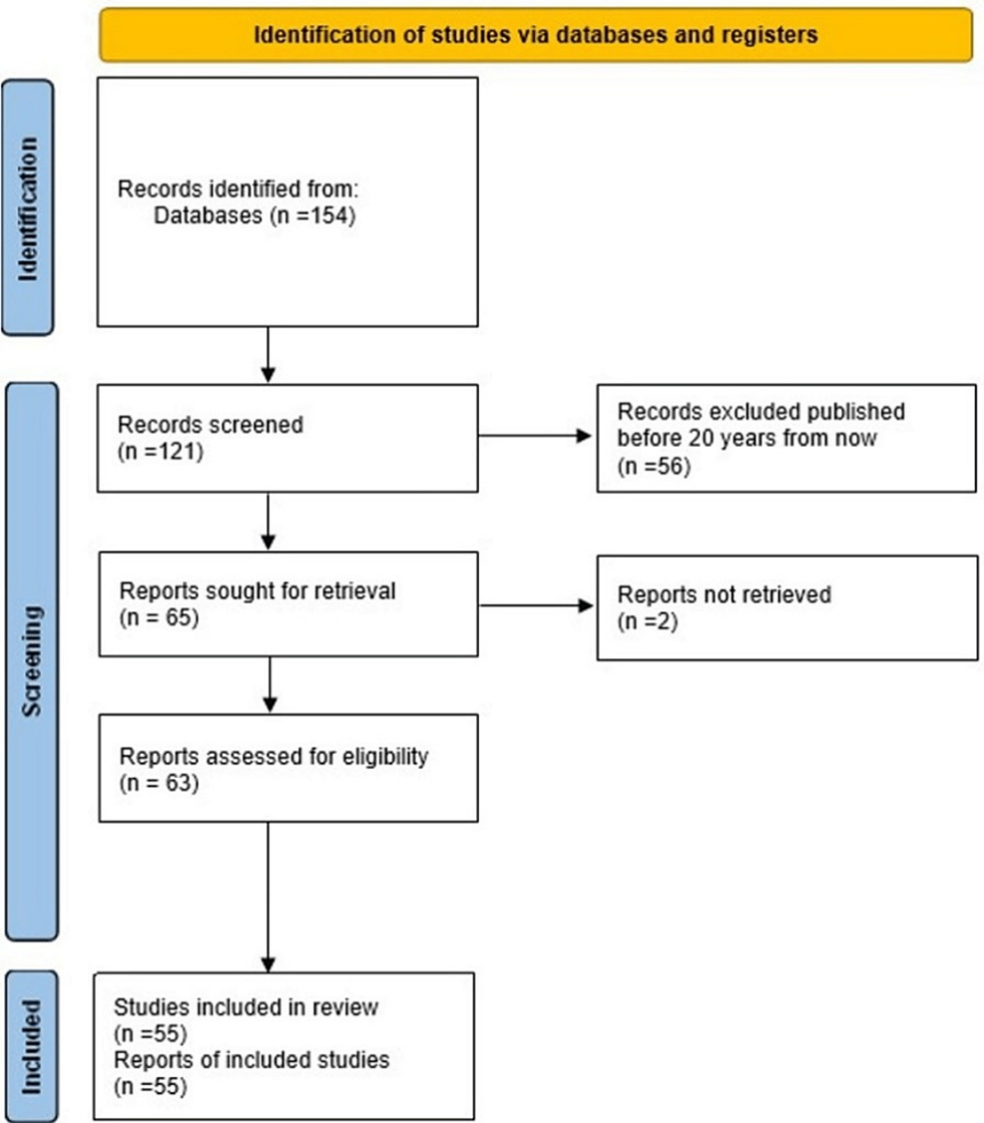
Preferred reporting items for systematic reviews and meta-analyses (PRISMA) flow diagram shows study selection process

Fournier’s gangrene (FG) is a surgical emergency condition in which extensive necrosis of soft tissue and fascia around the genital and perineal regions [1,2]. Escherichia coli and Bacteroides fragilis are the two main organisms associated with Fournier's gangrene. They work in concert to release collagenase enzymes that cause extensive tissue damage at a rate of one inch per hour and can spread to the anterior abdominal wall and vital organs, causing multi-organ failure and septic shock [3]. Diabetes has been a significant risk factor, reported in several cases. There are other comorbid conditions associated with FG, such as HIV, advanced age, alcoholism, smoking, obesity, atherosclerosis, and malnutrition [3,4]. Pain and discomfort in the perineum and genital area are the typical patient complaints at the initial presentation of FG. In one of the studies, 79% of patients reported scrotal swelling as their initial symptom. Fever, palpitation, purulent discharge, and crepitus were also reported. [5]. SGLT2-inhibitors used to treat type II diabetes mellitus can potentially cause necrotizing fasciitis in the perineum, but they have other side effects too. While SGLT2-inhibitors are one of the major anti-diabetic agents used and have significant cardio-protective and renal-protective qualities, this rare side effect should be considered by all physicians when initiating this treatment regimen. Although rare, there have been 55 published cases of necrotizing fasciitis in those who were administered SLT2-inhibitors from March 2013 to January 2019 [1]. This incited the US Food and Drug Administration (FDA) and European Medicines Agency (EMA) to issue a warning about these potential risks for those using SGLT2i-inhibitors in patients diagnosed with diabetes [2]. In this review article, we will review the current literature regarding the risks of FG in relation to initiating SGLT2 inhibitors (Figure 1).

## Review

SGLT-2 inhibitors and mechanism of action and its side effects 

Sodium-glucose cotransporter-2 (SGLT-2) inhibitors are commonly orally administered glucose-lowering drugs for adult patients with type-2 diabetes mellitus (T2DM). It is a relatively new subtype of the oral antidiabetic drug, which was first introduced in 2013 [6]. The sodium-glucose cotransport protein regulates the transport of sodium and glucose in intestinal cells, renal tubular cells, pancreatic alpha cells, the cerebellum, pulmonary cells, skeletal muscle, etc. [7,8]. SGLT-1 is found in enterocytes, kidney epithelial cells, lungs, and cardiac cells that control the transport of glucose and galactose, whereas SGLT-2 is mainly located in proximal renal tubular cells, pancreatic cells, and the cerebellum [7,8]. SGLT-2 inhibitors act predominately on the sodium-glucose co-transporter protein-2 in proximal renal epithelial cells in the kidney [6,9]. In normal circumstances, all filtered glucose is absorbed into the circulation and a minimal amount of glucose is excreted in the urine. Generally, the SGLT-2 co-transporter, located within the S1 segment of the renal proximal tubule, is responsible for the reabsorption of most of the glucose from the proximal tubule back into the systemic circulation. The rest of the glucose is reabsorbed by the SGLT-1 cotransporter in the S2 and S3 segments of the distal tubule [6,7,10]. SGLT-2 inhibitors prevent the function of this SGLT-2 co-transporter specifically. Hence, it lowers the blood glucose level and simultaneously increases urinary glucose excretion [6]. Not only is this drug effective in the reduction of blood glucose levels, but it has also been found that this group of drugs has a significant impact on lowering body weight through its glycoretic effect and better control of systemic hypertension [10].

**Figure 2:**
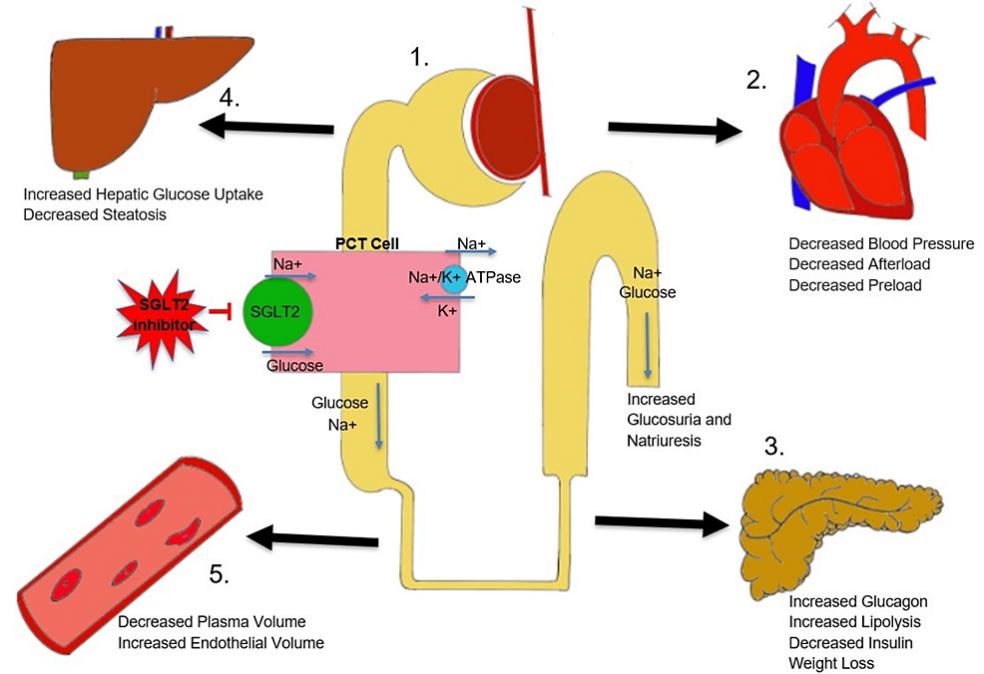
Mechanism of action of SGLT-2 inhibitors and their effects in various organs (1) Nephron, (2) heart, (3) pancreas, (4) liver, and (5) blood vessel

Several clinical trials have shown that this group of drugs has a better cardiovascular outcome in terms of multiple hospitalizations and mortality associated with heart failure [10]. Not only that, but it also provides an additional benefit to patients with chronic kidney disease (CKD) via its renoprotective effect [10]. Therefore, these drugs are regarded as the best choices for T2DM in CKD and heart failure patients (Figure 2) [9].

The FDA and the European Medicine Agency-approved SGLT-2 inhibitors include canagliflozin, dapagliflozin, ertugliflozin, and empagliflozin. Since these drugs increase urinary glucose excretion, expected side effects include glycosuria or polyuria [10]. Recently, the FDA warned about the serious genital infections associated with the initiation of these SGLT-2 inhibitors [9]. Fournier's gangrene (FG) is the specific term used for the development of a ra threatening necrotizing fasciitis of the perineum. It is also one of these infections that can specifically affect the perianal and genital areas of patients using these medications [9,11]. Although few case reports have been reported to explore the relationship between SGLT-2 inhibitors and Fournier's gangrene, systematic analysis has yet to be done [9].

Fournier’s gangrene and its association with SGLT-2 inhibitors: pathogenesis

Fournier's gangrene is a rare necrotizing fasciitis localized within the perineum and genitourinary region. FG is an aggressive infection that spreads rapidly and affects the tissues surrounding the muscles, nerves, fat, and blood vessels of the perineum, resulting in multi-organ dysfunction, septic shock, and even death [12]. Therefore, early recognition of necrotizing infection is critical. Infections are usually multi-microbial. The most common infectious agents include *E. coli*, *K. pneumoniae*, *B. fragilis*, and *Staphylococcus aureus* [13]. The most common comorbid risk factors include T2DM, alcohol misuse, and immunosuppression. During the course of the disease, the patient may experience symptoms of tenderness, redness, or swelling of the genitals or the area from the genitals back to the rectum, and have a fever above 100.4 °F or a general feeling of being unwell [14].

The pathophysiology of T2DM places patients at a higher risk of urinary tract and genital infections, despite the fact that the exact pathophysiologic processes of FG development are unknown. Glucosuria, immunological dysfunction, and enhanced bacterial adhesion to uroepithelium all contribute to this increased risk [15]. Increased urine glucose concentrations caused by SGLT2 inhibitors create a favorable environment for urinary and genital infections, which are a precursor to fasciitis gangrene development. With this infection, bacteria can enter into deeper tissue through an open cut or abrasion, then subsequently infect the tissue beneath the skin and destroy it. Bacteremia is thought to be the first step in the development of fascia necrosis, as it triggers a cytokine cascade that damages the endothelium, which presumptively triggers a coagulation cascade. This perpetuates the inhibition of fibrinolysis and the formation of disseminated micro thrombosis in vessels feeding the fascia via thromboplastin. Damage to the endothelium also causes fluid extravasation, inducing tissue swelling and leukocyte infiltration, all of which contribute to fascia ischemia and necrosis [16]. First and foremost, if gangrene is proven or even suspected, SGLT2 inhibitor therapy should be discontinued promptly. Simultaneously, gangrene therapy must begin quickly and may involve antibiotics and surgical debridement, depending on the clinical presentation.

Discussion of cases associated with Fournier’s gangrene and SGLT-2 inhibitors

At the end of August 2018, the U.S. Food and Drug Administration (FDA) fitted out a black box warning with reference to a possible association between Fournier’s gangrene and SGLT-2 inhibitors [17]. A total of 55 patients (39 men and 16 women) who were diagnosed with Fournier’s gangrene were reported to the FDA Adverse Event Reporting System (FAERS) from March 2013 up to January 2019 [17,2]. This was quite significant as there were only 19 cases of FG reported among patients who were taking other antidiabetic medications within the last 35 years [2,18]. Of these 55 patients, 3 patients died, with 25 of these patients requiring multiple debridement surgeries [19]. Additionally, the majority of these patients suffered from severe life-threatening complications, including acute kidney injury, diabetic ketoacidosis, and sepsis [19]. The mean duration of developing FG from starting an SGLT2 inhibitor was approximately nine months (ranging from 5 days to 49 months) [18-21].

Studies have shown that the incidence of FG is roughly 1.6 per 100,000 among males (3.3/100,000 among males aged between 50 and 79 years) [2,22,23] and 0.25 per 100,000 among females in the US. The cause of fewer incidents among females may be due to better drainage of the perineal region by vaginal secretion along with a confounding physician’s lack of awareness of FG symptoms in women [24]. Though fewer in number, the severity of the disease and case fatality is higher among females than males [22]. A study in Japan showed the same concern that female patients have a higher case fatality rate [22]. Though the incidence rate is low, studies have shown 7.5% of patients die from FG due to complications [24].

In Sweden, the first case of FG was reported in 2016. A literature review was performed among eight patients who were diagnosed with FG after starting an SGLT2 inhibitor. Of these eight patients, five were being treated with dapagliflozin, two patients were receiving empagliflozin; and one patient was receiving canagliflozin. The patients' ages ranged from 39 to 72, and six out of eight of the patients were obese. Additionally, four out of eight patients were smokers. The development of FG from the initiation of SGLT2 inhibitors varied from 10 days to six years, indicating significantly varied [17].

Another review/study showed that SGLT2 inhibitors were first launched in India in April 2015. From April 2015 to July 2018, 23 FG cases were identified. All the patients were males who were in their 50s and 60s. Of those, 70% had T2DM. All the patients required surgical intervention (the severity index was 5.08 ± 4.6) and three of them needed surgery more than once. Five patients needed to be shifted to the ICU, and three patients died from multiple organ dysfunction from sepsis [25].

Other than literature reviews, it is important to scrutinize the voluntary reports to have an overall better comprehension of the true incidence and prevalence of FG. The FAERS database has records of the suspected adverse outcomes reported by consumers, health professionals, and manufacturers. According to one study, 319 FG cases were reported by healthcare professionals, and among them, 43.00% were submitted by physicians. Consumers reported only 193 (35.61%) cases. Studies have also shown that the number of reported FG cases has jumped from 1 in 2014 to 407 in 2019, especially after the FDA warning in 2018. An increasing number of diabetes patients and the increasing popularity of using SGLT2 inhibitors may contribute to this huge escalation [24]. In this study, patients were also taking other concomitant drugs. Among them, drugs used for cardiovascular disorders are 34.84%, metformin is 34.81%, and insulin is 31.01%. The average time of FG onset was 293 days (ranging from 0 to 1365 days) among those who were taking only SGLT2 inhibitors and 373 days (ranging from 0 to 1696 days) among patients who were taking SGLT2 inhibitors along with other medications. Nonetheless, the data were very small to draw a conclusion [24].

By soliciting disproportionality analysis and Bayesian analysis in SGLT2 inhibitors and FG incidents in the FAERS database from January 2004 to September 2019 [24], studies have shown that among 542 FG patients who were taking SGLT2 inhibitors, Empagliflozin was linked with the highest number of FG reports (overall 232), followed by canagliflozin and dapagliflozin (199 and 108 cases, respectively) [24]. The results show that empagliflozin plus metformin had the strongest interconnection with FG, with higher ROR (54.79, 95% two-sided CI 31.56 to 95.12), PRR (53.36, χ2 666.70), IC (5.46, IC025 4.75), and EBGM (44.02, EBGM05 39.16) values than other SGLT2 inhibitors [24]. Three hundred ninety-one patients had to be admitted to the hospital (72.14%) and 26 patients died (4.81%) [24]. Till 2019, only three FG cases were found with ertugliflozin [24], however, this may be because of its limited time on the market [18,21]. But among all the SGLT2 inhibitors, ertugliflozin showed the shortest FG onset time (223 days) [24]. So far, no data regarding FG in association with dapagliflozin/saxagliptin, ertugliflozin/metformin, ertugliflozin/sitagliptin, ipragliflozin, luseogliflozin, or tofogliflozin were found [24].

When it comes to risk factors and comorbidities, a number of conditions are of concern; among them, pre-existing diabetes (20-70%) is the most concerning factor [22,24]. Studies have shown that in controlled or uncontrolled diabetes, the risk of developing FG is three times higher in diabetic patients [19,26]. Another significant concern is alcohol use (25-50%) [22]. Multiple case reports have been submitted where the significant comorbidity was obesity [18,25]. Other conditions include male sex, age [2], hypertension [23], smoking [9], end-stage renal disease, liver failure, immunocompromised conditions (including but not limited to HIV, inflammatory bowel disease, psoriasis, and malignancy), and any vascular disease [9,22,26]. As organisms need a portal to enter into host tissue, any infection [27], trauma, surgery, piercing, implants, intravenous drug usage, and any iatrogenic procedures in the genital area can be suggested as precipitating factors too [21]. According to some studies, the rate of infection among a diabetic patient with a penile prosthesis implant is 6 times higher than a non-diabetic patient [21]. Radiotherapy can be another precipitating factor. So far, five case reports have been submitted where patients have developed FG during or after radiotherapy [28].

Diagnosis and management

Unfortunately for physicians, the diagnosis of FG is largely based on clinical findings; there are no specific imaging or laboratory studies that can rule in or rule out FG or any necrotizing soft-tissue infection (NSTI) for that matter. This indicates that detailed investigations with a high index of suspicion are crucial in patients with risk factors for FG development [22]. However, to track disease progression, illness prognosis or to predict mortality, laboratory studies have been shown to be useful [29].

To combat the high mortality and swift progression of FG, several algorithms and calculators have been developed to enhance disease recognition and reduce disease severity [22]. These tools utilize clinical findings, laboratory data, and imaging to further guide physicians on the necessity of specific interventions required to prevent rapid patient deterioration. These tools include the Fournier's Gangrene Severity Index (FGSI), Uludag Fournier Gangrene Severity Index (UFGSI), Age-Adjusted Charlson Comorbidity Index (ACCI), the Laboratory Risk Indicator for Necrotizing Fasciitis (LRINEC) score, the Combined Urology and Plastics Index (CUPI), and the Platelet Mass Index (PMI) Score [22,30-33]. However, the absence of any one physical examination feature (e.g., fever or hypotension) is not sufficient to rule out NSTI. If there is a high suspicion of necrotizing fasciitis based on the history and physical examination findings, early involvement via surgical debridement should never be delayed regardless of the results of any clinical scoring system [22,29,30].

While imaging alone is not sufficient to rule out an NSTI or FG, it is certainly useful in planning surgical management. Specifically, computed tomography (CT) imaging has a diagnostic sensitivity of 88.5% and a specificity of 93.3% for NSTIs [22,29,34]. CTs will often reveal fluid collections, subcutaneous emphysema, abscesses, fascial thickening, and fat stranding in the presence of NSTIs [22,29,35]. Additionally, point-of-care ultrasound (POCUS) can be useful for detecting subcutaneous thickening, air, and fascial fluid. Castleberg et al. reported POCUS to have a diagnostic sensitivity of 88% and a specificity of 93% for NSTIs, making this method of imaging comparable to CTs [36,37]. In regards to FG, the presence of intrascrotal air and fat stranding is specific enough to instigate surgical management to prevent irreversible loss of patient sexual or urogenital function [35-37].

Since a majority of FG cases appear to be polymicrobial, an effective antibiotic cocktail should include coverage for streptococcal, staphylococcal, and Gram-negative bacteria; coliforms; Pseudomonas; Bacteroides; and Clostridium. Furthermore, coverage of MRSA species is recommended either with vancomycin or linezolid in conjunction with a carbapenem or beta-lactamase inhibitor (Table 1) [22,38,39]. There has been recent literature recommending adding clindamycin to the regimen as it has some capability to suppress bacterial toxin production, which can mediate the release of cytokines [38]. Doxycycline has also been recommended for patients who have a history of recent water exposure. This places them at increased risk of *A. hydrophila* and can further propagate an NSTI if not mitigated [22]. In those with a possibility of a superimposing fungal infection, antifungals including amphotericin B or fluconazole should also be included in the treatment regimen. Lastly, hyperbaric oxygen therapy (HBOT) can be considered due to its bacteriostatic and bactericidal effects in patients who have just undergone surgery, however, it is not considered in the acute care of patients [39].

**Table 1:** Empiric antibiotic regimen for the treatment of Fournier's gangrene

Empiric antibiotic regimen for Fournier’s gangrene [22,38,39]
Carbapenem (Imipenem, meropenem, or ertapenem)
OR
Beta lactam-beta lactamase inhibitor (piperacillin-tazobactam or ampicillin-sulbactam)
PLUS
Clindamycin for its activity against Gram-positive organisms and anaerobes, as well as its antitoxin effects
PLUS
Vancomycin, daptomycin, or linezolid for activity against gram-positive organisms and MRSA
In patients with severe hypersensitivity to carbapenems or beta lactam-beta lactamase inhibitors, consider
Aminoglycoside
OR
Fluoroquinolone
PLUS
Metronidazole
In patients with salt or freshwater exposure, consider adding:
Doxycycline
In patients with significant risk for fungal involvement, consider adding:
Amphotericin B or fluconazole

Emergent surgical debridement, broad-spectrum antibiotics, and appropriate resuscitation with intravenous fluids and vasopressor medications are the cornerstones of FG therapy [40,41]. This is in conjunction with controlling any other ongoing medical emergency that might have contributed to the development of FG. For example, a substantial number of patients with developing FG will present with diabetic ketoacidosis; since poorer glycemic control correlates with increasingly aggressive FG progression, strict control over blood sugars is required [41].

Severity and outcome

Given the extremely rapid progression and severe mortality rates of FG, there are several algorithms that have been developed to facilitate early detection and, subsequently, early treatment of FG. These tools include the Fournier's Gangrene Severity Index (FGSI), Uludag Fournier Gangrene Severity Index (UFGSI), Age-Adjusted Charlson Comorbidity Index (ACCI), the Laboratory Risk Indicator for Necrotizing Fasciitis (LRINEC) score, the Combined Urology and Plastics Index (CUPI) and the Platelet Mass Index (PMI) Score [22]. The LRINEC score utilizes levels of C-reactive protein, leukocytosis, hemoglobin, serum sodium, serum creatinine, and glucose to calculate the risk of a necrotizing soft tissue infection such as FG, with scores over 6 strongly indicating a necrotizing infection [22]. One study showed that patients with an average LRINEC of 5 have a significantly more favorable outcome than those with an average score of 10 [29]. This particular scoring system, however, only has an FG sensitivity of between 68% and 80% in patients in the emergency department setting; this lower value explains why several cases of necrotizing infections have been reported with low or zero points on this grading scale. However, there are studies that show that the degree of abnormality in these lab values, specifically white blood cell count, CRP levels, and platelet counts, can reflect the severity of the infection and predict patient outcomes [16].

The simplified Fournier’s Gangrene Severity Index is the most commonly used scoring system to determine the severity of FG-specific necrotizing infections developed by Lin et al. [25,31]. The sFGSI is a three-variable scoring system that predicts mortality and can determine whether a patient is high-risk or low-risk depending on their score early on in the pathogenesis of the disease. The use of the sFGSI is particularly useful in high-risk patients as early identification can lead to early intervention and therefore reduce the mortality rate. One study found that early intervention with the use of this scoring system was able to reduce mortality from 68.8% in those with later intervention to 23.8% in those with earlier intervention [41]. Earlier identification of FG can also decrease the severity of disease progression and reduce the potential body surface area affected [41].

The goal for patient outcomes in those with FG is to save the patient from sepsis and to minimize sexual and urinary dysfunction as much as possible [27]. While prompt patient admission and treatment with broad-spectrum antibiotics are indicated, the most definitive treatment of FG is aggressive surgical debridement of necrotic tissues [25,41]. Since the optimal window for surgery from the time of presentation is approximately 14.3 hours, earlier identification will allow for adequate activation of surgical management in a timely manner [31,41]. Conservative management with antibiotics alone has proven to be ineffective and can allow for sepsis and infection of soft tissues to worsen, and even increase the amount of body surface area affected [27,41].

**Figure 3:**
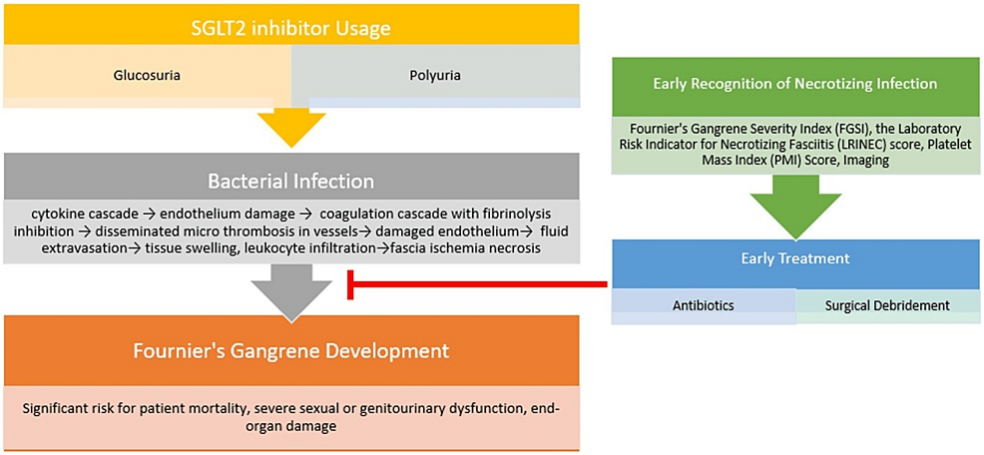
Schematic representing the pathogenesis of SGLT2 inhibitors in the development of Fournier’s gangrene

Those with a later diagnosis of FG and prolonged delays getting to the operating room required more aggressive surgical debridement and longer hospital stays [40,41]. One study saw that delays in treatment conferred up to an 88% mortality rate, with earlier detection reducing mortality by half, thus making surgical intervention one of the most crucial modifiable risk factors for the severity of FG [22] and making an earlier diagnosis in FG crucial to better patient outcomes. Oftentimes, it is necessary to return the patient to the operating table for repeat surgical debridement if there is enough evidence of continued disease progression. Attempting to salvage tissue to make future reconstruction easier is not recommended as this can lead to fulminant infection and increase the risk of debilitating disfigurement or patient death [16,27,40,41]. Additionally, in the management of FG patients, administration of SGLT2 inhibitors should be immediately stopped with alternative methods for strict glucose control being utilized (Figure 3) [27].

Things to consider before starting SGLT-2 inhibitors in diabetic patients

Type 2 diabetes mellitus (T2DM) is a condition resulting from a defect in the secretory function of pancreatic beta cells. Many medications are available to treat T2DM. SGLT-2 inhibitors are the newer group of oral antihyperglycemic medications which reduce blood sugar by increasing urinary glucose excretion. At present, T2DM is managed with either monotherapy with oral agents such as metformin or combination therapy with other groups of medicine. SGLT-2 inhibitors (empagliflozin, canagliflozin, dapagliflozin, etc.) are used with metformin to control blood glucose levels. Moreover, SGLT-2 inhibitors are part of the newest antidiabetic medication approved by the Food and Drug Administration [42].

Preinitiation Safety Screening

SGLT-2 inhibitors are the second-line drugs to control blood glucose levels along with other groups of antidiabetic medications [43,44]. The eligibility for prescribing SGLT-2 inhibitors depends on the type of diabetes, lifestyle, body habitus, hemodynamic status, and renal function status. Patients who are obese, hypertensive with type 2 DM, which is poorly controlled with monotherapy, and who are not willing to initiate insulin are the candidates for the start of SGLT-2 inhibitors [43-45]. Additionally, patients with T2DM with a heavy treatment burden, have contraindications to other common anti-diabetic agents, those susceptible to hypoglycemic events, or those who refuse to initiate insulin shots, are also candidates for initiating SGLT2-inhibitors [44,45].

Besides the many advantages of taking SGLT-2 inhibitors, patients with type 1 DM, peripheral arterial disease, bladder malignancy, and severe renal impairment are not suitable candidates to take this group of medications. In those cases, patients may develop euglycemic diabetic ketoacidosis, limb amputation, or acute kidney injury, respectively. Furthermore, it is also not recommended for hepatic impairment patients [42-44,46]. Table 2 below highlights eligible criteria for initiating SGLT2 inhibitor therapy [47-49].

**Table 2:** Eligible criteria to consider before starting SGLT-2 inhibitors and their effects DM: diabetes mellitus; HR: hazard ration; OR: odds ratio; BP: blood pressure; eGFR: estimated glomerular filtration rate; AKI: acute kidney injury; PVD: peripheral vascular disease; ACS: acute coronary syndrome

Eligible	Not eligible	Effect if not consider	HR/OR
Type 2 DM	Type 1 DM	Ketoacidosis	2.2 HR [47]
Hypertension	Hypotension	Lower BP	-
Obese	-	Weight loss	-
eGFR >30 ml/min/1.73 m^2^	End-stage renal disease and dialysis	AKI, electrolyte imbalance	-
Euvolemic	Dehydration	Orthostatic hypotension	-
-	PVD, neuropathy	Leg/foot amputation	1.97 HR [47]
-	Bladder cancer		3.87 OR [48]
T2DM with chronic HF	T2DM with ACS [50-52]	-	-

Susceptibility to Fournier's Gangrene in SGLT-2 Inhibitor Users

The most devastating adverse effect of SGLT-2 inhibitors is FG. Case reports prepared by Onder et al. and Kumar et al. have shown that the incidence of FG was more prevalent in uncontrolled T2DM patients who had poor genital hygiene due to morbid obesity [49,50]. Other factors are older age, immunosuppression, and local trauma. For this reason, special attention should be given to initiating SGLT-2 inhibitors in chronic uncontrolled diabetic patients who are morbidly obese to prevent FG formation [50,51,52].

Limitations

SGLT2 inhibitors are a relatively new class of antihyperglycemic agents found to be efficient in improving glycemic control. They are also authorized to be used in mitigating major adverse cardiovascular occurrences among adults with type-2 diabetes and have existing cardiovascular complications. Moreover, hospitalization rates for patients with heart failure were lower and kidney disease progression was reduced when SGLT2 inhibitors were administered. Diabetes patients are at high risk of developing gangrene, and one of the side effects of SGLT2 is also gangrene. But considering the evidence we have so far, the beneficial effects outweigh the negative side effects [28,53-55].

## Conclusions

FG exhibits extensive mortality and morbidity, especially in diabetic patients, which warrants the importance of prompt diagnosis of it. For SGLT2 inhibitor users, it is crucial for all clinicians to consider FG as a complication given its rapidly progressing nature, though a strong causal relationship has not been established in the literature. Being a promising class of antihyperglycemic agents, SGLT2 inhibitors have become an appealing medication modality since they have an advantageous cardiac and renal outcome. Significant microvascular complications and relative immunosuppression in DM could be blamed for the resulting FG, which can roughly be potentiated by SGLT2 use. This life-threatening necrotizing infection of the perineum in SGLT2 users demands further research to validate whether this medication is directly causing or only provoking the condition.
